# Anti-RNP Antibody: A Potential Novel Predictor for Osteonecrosis in Systemic Lupus Erythematosus

**DOI:** 10.3389/fmed.2022.847875

**Published:** 2022-04-11

**Authors:** Jiangbiao Xiong, Gang Wang, Tian Xu, Ren Liu, Shujiao Yu, Yan Wang, Rui Wu

**Affiliations:** ^1^Department of Rheumatology and Immunology, The First Affiliated Hospital of Nanchang University, Nanchang, China; ^2^Department of Rheumatology and Immunology, Gaoan Hospital of Traditional Chinese Medicine, Gaoan, China

**Keywords:** systemic lupus erythematosus, osteonecrosis, risk factor, autoantibodies, anti-RNP antibody

## Abstract

**Objective:**

To explore risk factors for developing osteonecrosis in patients with systemic lupus erythematosus (SLE).

**Methods:**

Twenty-six SLE patients with osteonecrosis from January 2018 to December 2019 were described. Fifty SLE patients without osteonecrosis were selected as controls from the SLE database (total 2,680) of our hospital during the same period. Clinical manifestations and laboratory tests were recorded and analyzed, especially antibodies. Univariate and multivariate analyses were used to evaluate possible associated risk factors.

**Results:**

Twenty-six (3 male, 23 female) SLE patients with osteonecrosis were confirmed by X-ray and magnetic resonance imaging. The median course from SLE onset to osteonecrosis onset was 45 (range 2–302) months. Seven (27%) patients had a single joint involved and 19 (73%) patients had two or more joints involved. Besides, the incidence of femoral head osteonecrosis (FHON), knee ON, and humerus head ON were 85% (22/26), 27% (7/26), and 12%(3/26), respectively. The multivariate logistic regression analysis showed that the score of European Consensus Lupus Activity Measurement (ECLAM) at SLE onset [odds ratio (OR) 1.37; 95% confidence interval (CI) 1.07–1.75], a cumulative dose of prednisone above 10 g (OR 15.49; 95% CI 3.38–84.61), and positive of independent anti-RNP antibodies (OR 3.35; 95% CI 0.80–10.73) were significantly associated with osteonecrosis in SLE.

**Conclusion:**

The score of ECLAM at SLE onset, a cumulative dose of prednisone above 10 g, and positive anti-RNP antibodies are associated with osteonecrosis in SLE. Herein, we reported for the first time that anti-RNP antibodies were associated with osteonecrosis in SLE patients and might be a novel predictor.

## Introduction

Osteonecrosis (ON) is usually induced by abnormal microvascular circulation which is believed to be the result of mechanical vascular interruption, intravascular occlusion, and extravascular compression (1). However, there are various reasons for ON in systemic lupus erythematosus (SLE) which leads to disability and affects the quality of life seriously in SLE patients. The first case of an SLE patient with ON (SLE-ON) was reported by Dubois and Cozen (2). But so far, the etiology and pathogenesis of ON in lupus remain unclear and treatment is challenged. It was initially recognized that glucocorticoids (GCs) are the main factor for lupus osteonecrosis (3). However, studies showed that other related risk factors, including gene susceptibility, lupus activity, vasculitis, Raynaud's phenomenon, hyperlipidemia, thrombophlebitis, and antiphospholipid antibodies (APL) also played a potential role in lupus osteonecrosis, although some results remained inconsistent (4–9). Early diagnosis and intervention are essential for improving the outcome of ON. It is necessary to screen out the possible risk factors for ON in SLE, which could help clinicians to identify susceptible individuals and provide prophylactic suggestions. To this point, we conducted a case-control study to analyze the clinical features and immunologic patterns of SLE patients with ON and explore the possible risk factors.

## Materials and Methods

This was a cross-sectional case-control study that SLE patients with or without ON were recruited from the SLE database (total 2,680) of the First Affiliated Hospital of Nanchang University during the period from January 2018 to December 2020 who fulfilled the Systemic Lupus International Collaborating Clinics classification criteria for systemic lupus erythematosus (10). Written informed consents were obtained from all patients. ON patients were confirmed by the X-ray and magnetic resonance imaging (MRI). Patients with ON were matched with patients without ON by age, sex, and body mass index(BMI) in a ratio of 1:2. Patients who overlapped with other connective tissue diseases were excluded. Disease activity was assessed according to the European Consensus Lupus Activity Measurement (ECLAM) (11). Anti-nuclear antibodies (ANA) were detected by indirect immunofluorescence (IIF). Anti-dsDNA antibodies, anticardiolipin antibodies (ACA), and anti-β2-glycoprotein I antibodies (β2GP1) were tested by ELISA while lupus anticoagulant (LAC) was tested by a dilute Russell's viper venom time with confirmatory studies. Euroline ANA profile (IgG) (EUROIMMUN Medizinische Labordiagnostika AG, Germany) was used to test 14 specific antibodies, including anti-nRNP /Sm antibodies, anti-Sm antibodies, anti-SSA antibodies, anti-SSB antibodies, anti-Ro-52 antibodies, anti-Scl-70 antibodies, anti-PM-SCL antibodies, anti-Jo-1 antibodies, anti-CENP-B antibodies, anti-PCNA antibodies, anti-ANuA antibodies, anti-Histone antibodies, anti-Rib-P antibodies, and AMA-M2 antibodies. Clinical features, laboratory indices, and treatment were collected and compared between the ON and the control group. The ethics committee of the First Affiliated Hospital of Nanchang University has confirmed that no ethical approval is required for this retrospective study.

SPSS 20.0 was used for analyses. Categorical data were analyzed by Chi-square test or Fisher exact test and numerical data by Student's *t*-test or Mann-Whitney test. A multivariate logistic stepwise regression was performed to identify factors associated with ON. A *P* < 0.05 was considered to indicate statistical significance.

## Results

**Figure 1:**
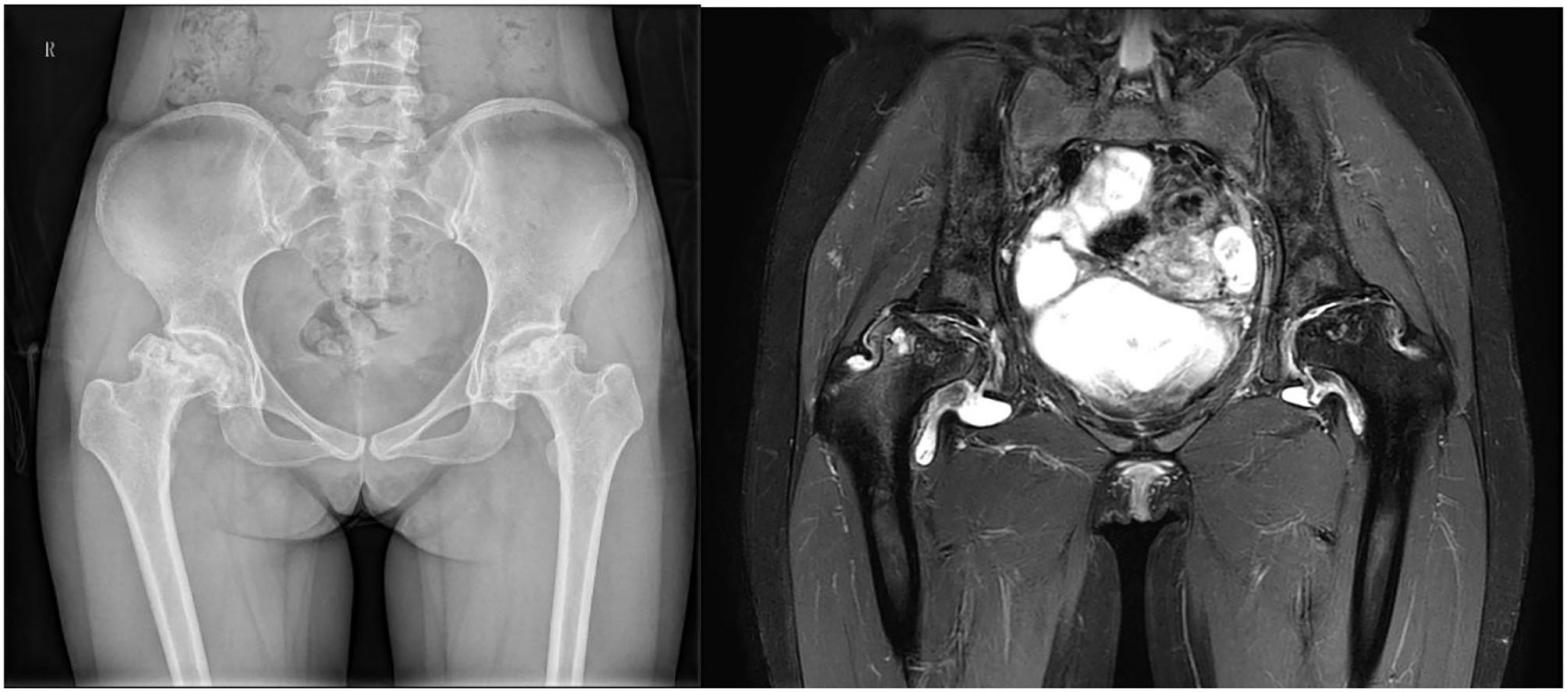
A 35-year-old woman with a history of SLE for 3.5 years was diagnosed with FHON.

**Figure 2:**
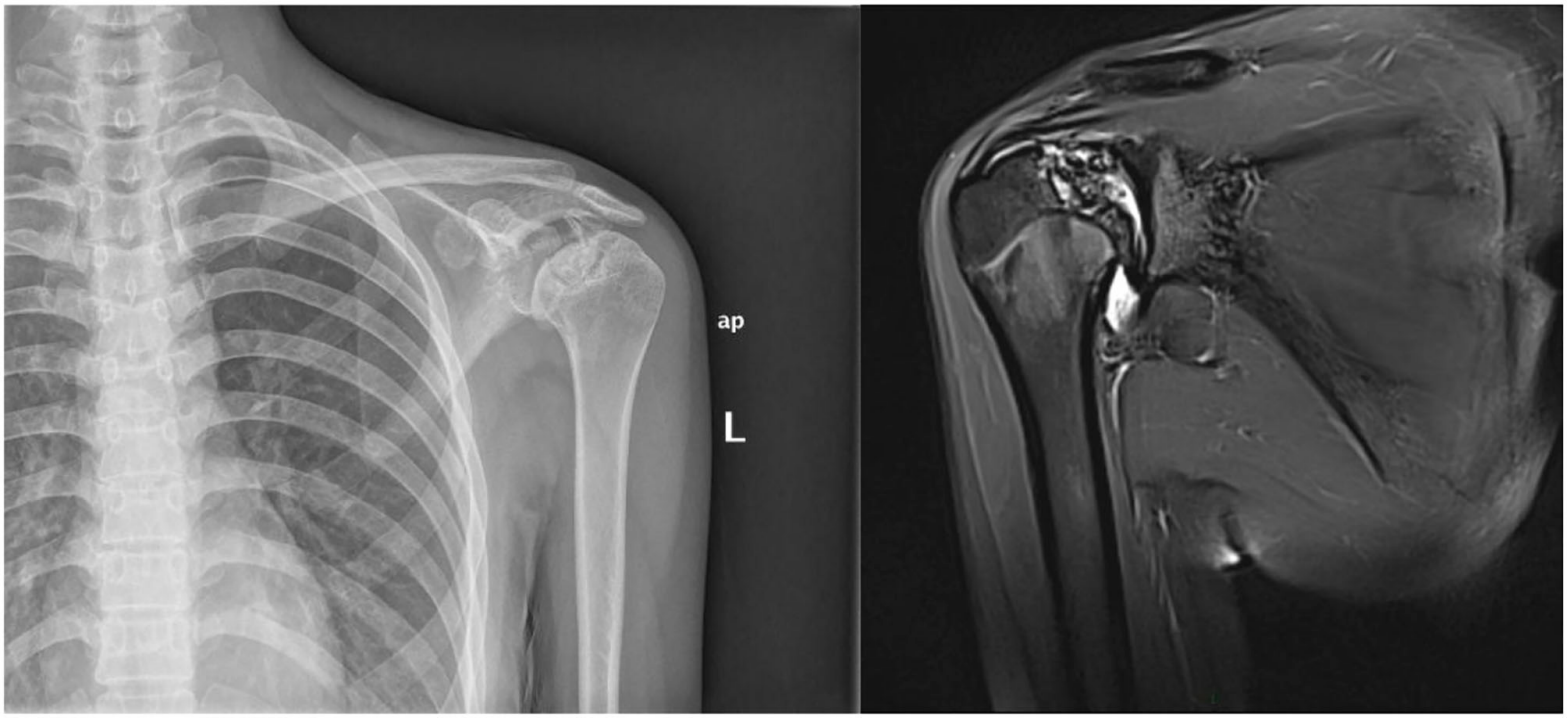
A 20-year-old woman with a history of SLE for 2 years was diagnosed with humerus head ON.

**Figure 3:**
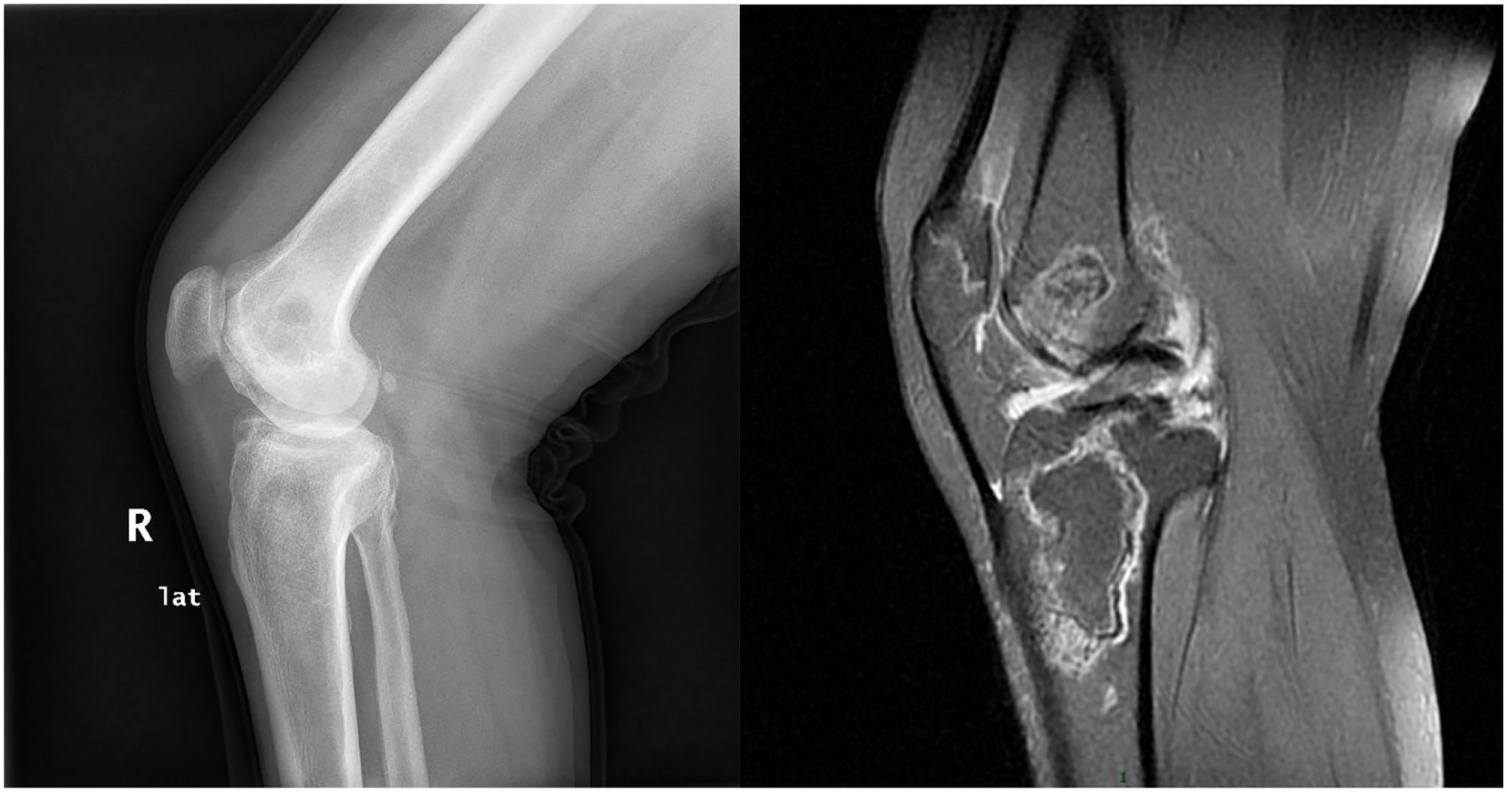
A 48-year-old woman with a history of SLE for 7 years was diagnosed with knee ON.

Twenty-six SLE patients (23 female, 3 male) were diagnosed with ON from January 2018 to December 2020, and 50 SLE patients excluded from ON were enrolled. All the ON patients had no history of alcohol abuse, smoking, oral contraceptives, or trauma. The clinical and laboratory characteristics of patients with ON were summarized in Table 1. More than half of patients developed ON in 45 months from SLE onset (Figures 1–3). A higher score of ECLAM was shown in the SLE-ON group than the control group (*P* < 0.05). Age at SLE onset was significantly younger than the control group (*P* < 0.05). Nephritis was more common in the SLE-ON group than the control group (*P* < 0.05) while no significant difference was shown in terms of rash, arthritis, cutaneous vasculitis, and neuropsychiatric disorders. Thrombosis events happened in 2 patients with ON. No significant difference in comorbidities of hypertension, diabetes, and hyperlipidemia was observed between the SLE-ON group and the control group.

**Table 1:** Characteristics of patients in the SLE-ON group and control group.

**Characteristics**	**SLE-ON (*n* = 26)**	**Control (*n* = 50)**	***P* value**
Male/female, *n*	3/23	2/48	0.216
Mean age at SLE onset, years (SD)	26.3 (9.0)	34.2 (15.1)	0.017^*^
Disease duration, years	6.0 (6.7)	5.4 (5.1)	0.661
Mean age at ON onset, years (SD)	32.5 (11.0)	-	-
ECLAM at SLE onset (SD)	4.7 (2.0)	2.8 (1.7)	<0.001^*^
ECLAM at ON onset (SD)	2.9 (1.1)	-	-
BMI, kg/m^2^ (SD)	21.5 (3.7)	21.2 (2.4)	0.680
Alopecia, *n* (%)	6 (23.1)	13 (26.0)	0.506
Raynaud's phenomenon, *n* (%)	5 (19.2)	3 (6.0)	0.085
Oral ulcers, *n* (%)	3 (11.5)	1 (2.0)	0.113
Rash, *n* (%)	7 (26.9)	13 (26.0)	0.569
Arthritis, *n* (%)	10 (38.5)	26 (52.0)	0.190
Cutaneous vasculitis, *n* (%)	4 (15.4)	1 (2.0)	0.081
Nephritis, *n* (%)	9 (34.6)	3 (6.0)	0.011^*^
Hematological disorder, *n* (%)	12 (46.2)	17 (34.0)	0.215
Neuropsychiatric disorder, *n* (%)	2 (7.7)	0 (0.0)	0.108
ESR, mm/h (SD)	39.2 (12.1)	33.6 (15.2)	0.108
D-dimer, mg/L (SD)	1.1 (1.8)	0.5 (1.0)	0.078
Treatment before ON			
Cumulative dose of PSL, g (SD)	21.3 (17.3)	14.1 (10.5)	0.028^*^
Daily dose of PSL, mg (SD)	19.4 (11.6)	11.3 (7.6)	<0.001^*^
Largest daily dose PSL, mg (SD)	56.6 (19.9)	39.7 (10.5)	<0.001^*^
CTX, *n* (%)	15 (57.7)	22 (44.0)	0.516
MMF, *n* (%)	19 (73.1)	29 (58.0)	0.211
MTX, *n* (%)	7 (26.9)	7 (14.0)	0.143
CsA, *n* (%)	4 (15.4)	5 (10.0)	0.268
TAC, *n* (%)	1 (3.9)	4 (8.0)	0.439
HCQ, *n* (%)	24 (92.3)	41 (82.0)	0.150
Comorbidities			
Hyperlipidemia, *n* (%)	9 (34.6)	17 (34.0)	0.957
Hypertension, *n* (%)	6 (23.1)	4 (8.0)	0.137
Diabetes mellitus, *n* (%)	1 (3.9)	1 (2.0)	0.570

SLE, systemic lupus erythematosus; ON, osteonecrosis; SD, standard deviation; ECLAM, European Consensus Lupus Activity Measurement; BMI, body mass index; ESR, erythrocyte sedimentation rate; PSL, prednisolone; CTX, cyclophosphamide; MMF, mycophenolate mofetil; MTX, methotrexate; CsA, cyclosporine; TAC, tacrolimus; HCQ, hydroxychloroquine, ^*^p < 0.05.

A higher prednisolone dose was shown in the SLE-ON group. Seventeen (65%) patients with ON were treated with prednisolone (PSL) at a cumulative dose of more than 10 g. All the patients with ON had been treated with immunosuppressant agents (ISA), such as cyclophosphamide (CTX), methotrexate (MTX), mycophenolate mofetil (MMF), cyclosporine (CsA), tacrolimus (TAC), and hydroxychloroquine (HCQ). No significant difference in ISA administration was shown between the SLE-ON and control groups. Seven (27%) patients had a single joint involved and 19 (73%) patients had two or more joints involved. The incidence of femoral head osteonecrosis (FHON), knee ON, and humerus head ON were 85% (22/26), 27% (7/26), and 12% (3/26), respectively.

The frequency of the autoantibodies is presented in Table 2. No significant difference was found in the titer of ANA (IIF) between the SLE-ON and control group. Anti-dsDNA and other 14 specific antibodies were found insignificantly different between two groups. The frequency of independent anti-RNP antibodies (with negative anti-Sm antibodies) was significantly higher in the SLE-ON group (*p* = 0.014).

**Table 2:** Immunologic features in the SLE-ON group and control group.

**Immunological tests**	**SLE-ON (*n* = 26)**	**Control (*n* = 50)**	***P* value**
ANA (IIF), *n* (%)	26 (100)	50 (100)	0.933
Titer			
100–320	15	26 (52)	
320–1,000	4	10 (20)	
>1,000	7	14 (28)	
APL, *n* (%)	8 (30.8)	7 (14.0)	0.143
ACA, *n* (%)	2 (7.7)	4 (15.4)	0.667
anti-β2GP1, *n* (%)	4 (15.4)	4 (15.4)	0.249
LAC, *n* (%)	6 (23.1)	5 (19.2)	0.111
Anti-dsDNA, *n* (%)	13 (50%)	19 (38.0)	0.531
ANAs (IBT),*n* (%)			
anti-nRNP/Sm	18 (69.2)	19 (38.0)	0.072
anti-Sm	7 (26.9)	13 (26.0)	0.544
anti-RNP (nRNP/Sm+ and Sm-)	11 (42.3)	6 (12.0)	0.014^*^
anti-SSA	15 (57.7)	34 (68.0)	0.220
anti-SSB	3 (11.5)	10 (20.0)	0.271
anti-Ro52	12 (46.2)	27 (54.0)	0.321
anti-Scl-70	1 (3.8)	3 (6.0)	0.575
anti-PM-SCL	1 (3.8)	1 (2.0)	0.572
anti-Jo-1	0 (0)	1 (2.0)	0.657
anti-CENP-B	1 (3.8)	1 (2.0)	0.572
anti-PCNA	0 (0)	1 (2.0)	0.657
ANuA	5 (19.2)	11 (22.0)	0.509
anti-Histone	9 (34.6)	14 (28.0)	0.369
anti-Rib-P	11 (42.3)	14 (28.0)	0.154
AMA-M2	0 (0)	5 (10.0)	0.112
Low C3 (g/L), *n* (%)	20 (76.9)	29 (58.0)	0.102
Low C4 (g/L), *n* (%)	19 (73.1)	30 (60.0)	0.258

SLE, systemic lupus erythematosus; ON, osteonecrosis; ANA, anti-nuclear antibodies; IIF, indirect immunofluorescence; APL, antiphospholipid antibodies; ACA, anticardiolipin antibodies; β2GP1, β2-glycoprotein I; LAC, lupus anticoagulant; IBT, immunoblotting test, ^*^p < 0.05.

The multivariate logistic regression analysis showed that the score of ECLAM at SLE onset [odds ratio (OR) 1.37; 95% confidence interval (CI) 1.07–1.75], a cumulative dose of PSL above 10 g (OR 15.49; 95% CI 3.38–84.61), and positive of independent anti-RNP antibodies (OR 3.35; 95% CI 0.80–10.73) had significant associations with SLE-ON (Table 3).

**Table 3:** Multivariate logistic regression analysis of parameters for ON.

**Parameters**	**OR (95% CI)**	***P* value**
Mean age at SLE onset	0.90 (0.83–0.97)	0.270
ECLAM at SLE onset	1.37 (1.07–1.75)	0.048^*^
Anti-nRNP/Sm	1.91 (0.31–6.48)	0.084
Anti-RNP (nRNP/Sm+ and Sm-)	3.35 (0.80–10.73)	0.021^*^
Nephritis	4.26 (1.08–15.91)	0.059
Cumulative dose of PSL>10g	15.49 (3.38–84.61)	0.012^*^

SLE, systemic lupus erythematosus; ON, osteonecrosis; OR, odds ratio; CI, confidence interval; ECLAM, European Consensus Lupus Activity Measurement; PSL, prednisolone, ^*^p < 0.05.

## Discussion

Diagnosis of ON in SLE is always delayed because of insidious symptoms. Most patients show mild arthralgia without swelling and limited movement in the early stage of ON (12). In this study, the MRI of all the patients at the time of being diagnosed with ON showed they were already at or above the second stage of ON (13). Moreover, ON is more likely to occur in young patients with SLE (12, 14). As was shown in this study, the mean age of SLE onset and the mean age of ON onset of the SLE-ON group were 26.3 years and 32.5 years, respectively. Reports have estimated the prevalence of ON in SLE patients ranging from 4 to 40% (15, 16). In our two-year SLE database of 2,680 patients, only 26 (1%) patients have been diagnosed with ON, which is lower than the literature, suggesting that there may be underdiagnosis of ON in our center.

For decades, GCs have been considered as an independent predictor of ON (17–20). This study improved that point by the discovery that a cumulative dose of GCs above 10 g, an average daily dose of GCs above 20 mg, and the largest daily dose of GCs above 40 mg are risk factors for ON. In addition, the use of GCs was more likely to be correlated with FHON than other parts of ON (12). Nevertheless, studies showed that inflammation and vascular endothelial cell injuries due to SLE also contributed to the occurrence of ON (21, 22). Lupus disease activity, vasculitis, and systemic damage including nephritis have been believed to be risk factors for early ON in some reports (5, 16), which is consistent with us. APL has also been proved as a risk factor for ON in some studies (23–25). However, neither ACA nor LA showed a significant association with ON in this study, although the prevalence of positive ACA and LA were higher in the ON group.

Among autoantibodies tested in this study, only positive anti-RNP/Sm with negative anti-Sm antibodies were found significantly associated with ON, suggesting that anti-U1RNP antibodies may be an independent risk factor. The exact role of anti-RNP antibodies in the pathogenesis of autoimmune diseases remains unclear. Literature shows anti-RNP antibodies are related with manifestations of vascular disorders, for example, Raynaud's phenomenon, via affecting endothelial cells (26, 27). Therefore, the appearance of independent anti-RNP antibodies suggests that endothelial injuries play a role in the pathogenesis of ON in SLE. It is well known that the positive independent anti-RNP antibodies are strongly associated with mixed connective tissue disease (MCTD) (28). However, it is interesting to note that the incidence of ON in MCTD is not as frequent as SLE. It is reported that the IgM type of anti-U1RNP antibodies is predominantly present in SLE than MCTD (29), which reveals that the different types of anti-U1RNP antibodies may have different pathogenicities in ON.

The study has several limitations. First, the evidence is limited for the case-control design. Second, the sample size of SLE patients with ON is not large enough. In the future, a prospective study with a larger sample will be needed for the verification of these findings.

In conclusion, the score of ECLAM at SLE onset, a cumulative dose of PSL above 10 g, and positive anti-RNP antibodies are associated with ON in SLE. Herein, we reported for the first time that anti-RNP antibodies were associated with ON in SLE and might be a novel predictor.

## Data Availability Statement

The raw data supporting the conclusions of this article will be made available by the authors, without undue reservation.

## Ethics Statement

Ethical review and approval was not required for the study on human participants in accordance with the local legislation and institutional requirements. Written informed consent to participate in this study was provided by the participants' legal guardian/next of kin. Written informed consent was obtained from the individual(s), and minor(s)' legal guardian/next of kin, for the publication of any potentially identifiable images or data included in this article.

## Author Contributions

JX and RW: study design and drafting of manuscript. TX, RL, YW, and GW: data collection and data analysis. All authors contributed to the article and approved the submitted version.

## Conflict of Interest

The authors declare that the research was conducted in the absence of any commercial or financial relationships that could be construed as a potential conflict of interest.

## Publisher's Note

All claims expressed in this article are solely those of the authors and do not necessarily represent those of their affiliated organizations, or those of the publisher, the editors and the reviewers. Any product that may be evaluated in this article, or claim that may be made by its manufacturer, is not guaranteed or endorsed by the publisher.
